# The Use of Match Statistics that Discriminate Between Successful and Unsuccessful Soccer Teams

**DOI:** 10.2478/v10078-012-0015-7

**Published:** 2012-04-03

**Authors:** Julen Castellano, David Casamichana, Carlos Lago

**Affiliations:** 1 University of the Basque Country (UPV/EHU), Department of Physical Education and Sport, Vitoria–Gasteiz, Spain.; 2 University of Vigo, Faculty of Education and Sports Sciences, Pontevedra, Spain.

**Keywords:** soccer, match analysis, performance indicators, discriminant analysis

## Abstract

Three soccer World Cups were analysed with the aim of identifying the match statistics which best discriminated between winning, drawing and losing teams. The analysis was based on 177 matches played during the three most recent World Cup tournaments: Korea/Japan 2002 (59), Germany 2006 (59) and South Africa 2010 (59). Two categories of variables were studied: 1) those related to attacking play: goals scored, total shots, shots on target, shots off target, ball possession, number of off-sides committed, fouls received and corners; and 2) those related to defence: total shots received, shots on target received, shots off target received, off-sides received, fouls committed, corners against, yellow cards and red cards. Discriminant analysis of these matches revealed the following: (a) the variables related to attacking play that best differentiated between winning, drawing and losing teams were total shots, shots on target and ball possession; and (b) the most discriminating variables related to defence were total shots received and shots on target received. These results suggest that winning, drawing and losing national teams may be discriminated from one another on the basis of variables such as ball possession and the effectiveness of their attacking play. This information may be of benefit to both coaches and players, adding to their knowledge about soccer performance indicators and helping to guide the training process.

## Introduction

A match analysis is commonly used in many sports and is viewed as a vital process that enables coaches to collect objective information which can be used to provide feedback on performance (Carling et al., 2005). As coaches are prone to making subjective judgments and may be unable to recall events reliably, they are increasingly turning to match analysis as a way of optimizing the training process of their players and teams (Hughes and Franks, 2004). The main aim of match analysis is to identify the strengths and weaknesses of one’s own team, thereby enabling the former to be further developed and the latter to be worked upon. Similarly, a coach analysing the performance of an opposing side will use the data to identify ways of countering that team’s strengths and exploiting its weaknesses (Carling et al., 2008).

Performance indicators in sport can be defined as the selection and combination of variables that define some aspects of performance and which help to achieve success (Hughes and Bartlett, 2002). These indicators constitute an ideal profile that can be used to predict future behaviour in a given sporting activity (O’Donoghue, 2005). In the context of soccer the World Cup is undoubtedly the greatest prize and it provides an opportunity to compare the best teams and players in the world. Usually, after a World Cup, successful teams set new trends in terms of training and playing style. Indeed, others will tend to imitate the tactics and play of winning teams, seeking to master those aspects of performance which are deemed to underlie their success (Hughes and Franks, 2005).

Other than in the historical field, however, there have been very few longitudinal studies of soccer play, and although the game has evolved considerably over the last fifty years (Kuhn, 2005) the style of play appears to have changed very little during the last decade if one considers the general playing style of teams competing at the World Cup (Castellano et al., 2008). Empirical match analysis of World Cups has generally focused on three main aspects:

- Goal scoring and patterns of build-up play leading to shots (Acar et al., 2009; Armatas and Yiannakos, 2010; Bate, 1988; Dufour, 1993; Ensum et al., 2005; Grant et al., 1999; Grèhaigne, 1998; Jinshan et al., 1993; Olsem, 1988; Sajadi and Rahnama, 2007; Starosta, 1988; Winkler, 1988; Hughes and Franks, 2005; Jones et al., 2004).
- The development of the whole process of attacking/defensive play. For example, research has sought to determine patterns of play (Castellano et al., 2007a) or identify playing styles in the following World Cups: Spain 1982 (Pollard et al., 1988), Italy 1990 (Partridge et al., 1993), USA 1994 (Hughes and Franks, 2005), Korea/Japan 2002 (Scoulding et al., 2004) and Germany 2006 (Castellano et al., 2007b; Rowlinson and O′Donoghue, 2009; Xu et al., 2007).
- Finally, some studies have related these aspects to the match result (winning or losing) (Hughes et al., 1988) in Italy 1990 (Bishovets et al., 1993; Yamanaka et al., 1993), France 1998 and Korea/Japan 2002 (Lawlor et al., 2003).

However, although these studies examined indicators of success in soccer, their results remain inconclusive due to certain limitations and/or methodological problems. For instance, some of these studies are based on small sample sizes and usually conduct a univariate analysis of the observed variable. These factors are likely to influence the results regarding team’s performance and thus may contribute to the differences found in existing studies.

The aim of the present study was therefore twofold. Firstly, and given the limitations of extant research, we sought to identify, by means of different multivariate analyses, the match statistics which best discriminated between winning, drawing and losing teams in the last three World Cups. Secondly, we examined how the performance profile of winning teams evolved during this period, the aim being to determine whether performance indicators have varied over the three tournaments studied.

## Material & methods

### Sample

The analysis was based on 177 matches played during the three most recent World Cups: Korea/Japan 2002, Germany 2006 and South Africa 2010. Although a total of 192 matches were actually played during these three tournaments we excluded those matches in which extra time was played. The final sample therefore comprised 59 matches from each of the three World Cups (equivalent to 92.2% of all matches played). The data was obtained from the FIFA website (http://fifa.com/worldcup/index.html). Their reliability was studied by coding five randomly-chosen matches and comparing the data obtained with those from the FIFA website. The resulting values of Cohen’s kappa (K) were between 0.93 and 0.97.

### Procedure

Two categories of variables were studied: those related to attacking play and those related to defence (Table 1). The following match statistics were gathered: goals scored, total shots, shots on target, shots off target, ball possession, off-sides, fouls, corners, yellow cards and red cards (both committed and received for the two teams).

### Statistical analysis

A descriptive analysis was first carried out. The homogeneity of variances was examined by means of the Levene’s test and an analysis of variance (ANOVA) was then used to determine which variables revealed differences between the three categories of teams (winning, drawing and losing); this was done first for the whole set of matches (n = 177) and subsequently for each of the three World Cups (South Africa 2010, Germany 2006 and Korea/Japan 2002).

Whenever a significant difference was found, we applied either the post hoc Bonferroni test or, if the variances were not homogeneous, the Dunnett’s T3 post-hoc test. A discriminant analysis was then performed in order to identify the variables which best discriminated between winning, drawing and losing teams (Ntoumanis, 2001). This was achieved by calculating the structural coefficients (SC), with values >0.30 being regarded as significant (Tabachnick and Fidell, 2007). All the statistical analyses were performed using *SPSS 16.0 for Windows*, with significance being set at *p*<0.05.

## Results

Table 2 shows the descriptive results derived from the match statistics for winning, drawing and losing teams. In the first group of variables (performance indicators of attacking play) the averages for winning teams were significantly higher than those of both drawing and losing teams for the following match statistics: *goals scored, total shots* and *shots on target*. However, on the variables *ball possession* and *fouls received* they were only higher than the averages of losing teams (*p<0.01*).

There were no differences between the three categories of teams for the variables *shots off target, off-sides committed* and *corners*. In the second group of variables (performance indicators of defence) the averages of losing teams were significantly higher than those of both winning and drawing teams for the match statistics *total shots received* and *shots on target*. However, on *fouls committed* and *red cards* they were only higher than the averages of winning teams (*p<0.01*). There were no differences between the three categories of teams on the variables *shots off target, off-sides received, corners against* and *yellow cards*.

Table 3 shows the descriptive results derived from the match statistics for winning, drawing and losing teams in each of the three World Cups studied. This separate analysis of each tournament revealed certain differences in the performance profile of teams. In the first group of variables (attacking play), winning teams had consistent averages across the three World Cups that were significantly higher than those of drawing and losing teams for the following match statistics: *goals scored* and *shots on target*. However, on the variable of *ball possession* in Germany 2006 and South Africa 2010, and on the *fouls received* and *corners* in Germany 2006 the averages of winning teams were only significantly higher than those of losing teams (*p<0.01*). On the *total shots* in Korea/Japan 2002 the averages of winning teams were significantly higher than those of losing teams (*p<0.01*). There were no differences between the three categories of teams for the variables *shots off target* and *off-sides committed*. In the second group of variables (defensive play) the averages of losing teams were significantly higher than those of winning and drawing teams for *total shots received* and *shots on target received* (as well as *red cards* in South Africa 2010). However, on the *fouls committed* in South Africa 2010 and *corners against* in Germany 2006 their averages were only higher than those of winning teams (*p<0.01*). There were no differences between the three categories of teams on the variables *shots off target, off-sides received* and *yellow cards*.

Table 4 presents the results of the discriminant analysis for all three World Cups. The discriminant functions correctly classified 91.4% of winning, drawing and losing teams, and both of the two discriminant functions obtained were significant (*p<0.05*). In the first discriminant function the variables with the greatest discriminatory power were *total shots* (SC = 0.36), *shots on target* (SC = 0.62), *total shots received* (SC = −0.37), and *shots on target received* (SC = −0.56).

Table 5 shows the structural coefficients (SC) derived from the discriminant analysis of each World Cup separately. Four of the discriminant functions obtained were significant (*p<0.05*). However, there were differences between the World Cups in regard to variables which showed the greatest discriminatory power. In South Africa 2010 the most discriminating variables were *total shots* (SC = 0.56 in Function 1 and 0.53 in Function 2), *shots on target* (SC = 0.53 in Function 1), *ball possession* (SC = 0.32 in Function 1) and *red cards* (SC = 0.31 in Function 1). In Germany 2006 the most discriminating variables were *shots on target* (SC = 0.65), *shots on target received* (SC = 0.51), *total shots received* (SC = 0.46), *total shots* (SC = 0.45) and *ball possession* (SC = 0.31). Finally, in Korea/Japan 2002 the most discriminating variables were *shots on target* (SC = 0.74), *total shots* (SC = 0.50) and *red cards* (SC = 0.32).

## Discussion

The aim of this study was to identify the performance indicators that best discriminated between winning, drawing and losing teams in three soccer World Cups (Korea/Japan 2002, Germany 2006 and South Africa 2010), and to determine whether the indicators that differentiated between successful and unsuccessful teams were repeated across these three tournaments. In this context, the study is the first to have applied a multivariate analysis to performance indicators of World Cup matches.

The results of the initial univariate analysis identified nine variables that differed between winning, drawing and losing teams (Table 2), while in the subsequent multivariate analysis only four variables were found to discriminate teams in relation to their performance (Table 4). When analysing the three World Cups as a whole the variables with the greatest discriminatory power were *total shots* and *shots on target* (both *made* and *received*), although this finding was not constant across the three tournaments when analysed separately. Previous studies of soccer World Cups (those held in 1998, 2002 and 2006) found similar patterns of play among teams (Castellano et al., 2007b; 2008), while the present study shows slight differences in the variables that discriminate between successful and unsuccessful teams competing in the last three World Cups.

In line with the present findings, Lago et al. (2010) also found that the variable *shots on target* had the greatest discriminatory power with regard to matches played in the Spanish league, and statistically significant differences in its value have been reported between top and middle/lower ranking teams in the same league (Lago-Ballesteros and Lago, 2010). The same variable has also been shown to be one of the best at discriminating between successful and unsuccessful teams in Italy (Rampinini et al., 2009), as well as between national sides in the 2002 World Cup (Lawlor et al., 2003). It would seem, therefore, that what best discriminates team performance is the number of shots on target, and not the total number of shots made. This is consistent with the findings of Szwarc (2004), who reported that winning teams made only four more shots overall than did less successful teams, but the effectiveness of their shots was three-fold greater. Similar results were found by Yamanaka et al. (1993) and Bishovets et al. (1993) for national sides competing in the 1990 World Cup in Italy, as well as by research that analysed the shot variable in World Cups (Hughes et al., 1988).

Ball possession is one of the most widely-studied performance indicators (Lago and Martin, 2007), although its relationship to team performance requires further clarification. In the present study, ball possession was not a discriminating variable when the three World Cups were analysed as a whole, but it did show discriminant power in the 2010 and 2006 World Cups when these were analysed separately. However, although ball possession was a variable that discriminated between winning and other teams in the 2006 World Cup, the values for the side that won that tournament, Italy, were not significantly different from those of their rivals (Balyan et al., 2007), which could be due to their style of play. These differences may indicate that teams are now beginning to give greater importance to ball possession. Research conducted in Spain for the 2008–09 season (Lago-Ballesteros and Lago, 2010) also found significant differences in ball possession between league leaders and mid-table teams, although this was not the case with respect to the bottom teams.

Taking into consideration the variables related to defensive play, differences were found (Table 5) for *shots received* (in the 2006 and 2010 World Cups)*, shots on target received* (in 2006 and 2010)*, corners against* (in 2010) and *red cards* (in 2002 and 2010). However, when the three tournaments were analysed as a whole the only variables that discriminated between successful and unsuccessful teams were *total shots* and *shots on target received* (Table 4). At all events, very few studies (Lago et al., 2010) have reported differences in the number of *red cards*, *off-sides received* and *crosses received* for club sides. The present study found no differences between winning, drawing and losing teams in any of the three tournaments studied as regards the number of *off-sides received*. Similarly, a recent study by Lago-Ballesteros and Lago (2010) found no defence-related variable that differed between top, mid-table and bottom teams in the Spanish league, although it should be noted that this study did not consider shots received by the opposing team as a defence-related variable.

In summary, the comparative analysis over time of the performance profiles associated with winning teams may not only reveal how playing styles evolve or new trends emerge, but also identify those variables (such as ball possession or shots on target) which are considered the most important in soccer today. This study has analysed match statistics related to the attacking and defensive play of winning, drawing and losing teams in three World Cup tournaments. It has also examined how the performance profile of winning teams changes over time, and has sought to identify the performance indicators that best discriminate between successful and unsuccessful teams. Perhaps, the prediction for the WC in Brazil is that possession of the ball and pass proficiency remain key issues for successful team performance. The results may be of use to coaches in terms of designing their training programmes, providing them with information about what attacking players need to achieve, and what needs to be avoided defensively, if a team is to increase its chances of winning. In this sense, the game models based on indirect styles seem to have more chance of success in the near future. The effectiveness of attacking play (in terms of shots on target) and ball possession appear to be the performance indicators that constitute the keys to success in today’s soccer.

## Figures and Tables

**Table 1:** Variables studied in the last three soccer World Cups

Variables related to	Variables or match statistics or performance indicators
Attacking play	*Goals scored, Total shots, Shots on target, Shots off target, Ball possession, Off-sides committed, Fouls received, Corners.*
Defence	*Total shots received, Shots on target received, Shots off target received, Off-sides received, Fouls committed, Corners against, Yellow cards, Red cards.*

**Table 2:** Descriptive results and univariate differences between winning, drawing and losing teams according to match statistics derived from the whole sample of matches played during the last three World Cups

	Winning (n = 139)	Drawing (n = 78)	Losing (n = 139 )	F	P
Variables related to attacking play					

***Goals scored***	**2.2 ± 1.2^ab^**	**0.9 ± 0.8^b^**	0.4 ± 0.6	135.81	0.000
***Total shots***	**14.2 ± 5.1^ab^**	11.3 ± 4.4	10.7 ± 4.4	21.26	0.000
*Shots off target*	7.1 ± 3.6	6.7 ± 3.3	6.7 ± 3.3	0.62	0.539
***Shots on target***	**7.1 ± 2.6^ab^**	4.5 ± 2.4	4.0 ± 2.2	62.15	0.000
***Ball possession (%)***	**51.6 ± 6.8^b^**	49.9 ± 5.8	48.5 ± 6.8	7.46	0.001
*Off sides committed*	2.9 ± 2.5	2.7 ± 2.4	2.5 ± 1.7	1.16	0.315
***Fouls received***	**18.1 ± 6.2^b^**	16.9 ± 4.7	15.9 ± 5.2	5.62	0.004
*Corners*	5.4 ± 2.9	4.9 ± 3.1	4.8 ± 2.8	1.71	0.182

Variables related to defence					

***Total shots received***	10.7 ± 4.4	11.3 ± 4.4	**14.2 ± 5.1^ac^**	21.50	0.000
*Shots off target received*	6.6 ± 3.3	6.8 ± 3.3	7.1 ± 3.6	0.78	0.457
***Shots on target received***	4.1 ± 2.6	4.5 ± 2.4	**7.1 ± 2.6^ac^**	52.99	0.000
*Off sides received*	2.6 ± 1.7	2.6 ± 2.2	3.0 ± 2.5	1.38	0.252
***Fouls committed***	16.1 ± 5.3	17.2 ± 4.6	**17.9 ± 5.2^c^**	4.62	0.010
*Corners against*	4.7 ± 2.8	5.0 ± 3.1	5.4 ± 2.9	1.54	0.216
*Yellow cards*	2.0 ± 1.4	2.2 ± 1.4	2.1 ± 1.3	0.76	0.469
***Red cards***	0.06 ± 0.3	0.1 ± 0.4	**0.2 ± 0.5^c^**	7.75	0.001

a Significantly different from drawing teams,

b Significantly different from losing teams.

c Significantly different from winning teams.

**Table 3:** Descriptive results and univariate differences between winning, drawing and losing teams according to match statistics from each of the three World Cups studied.

	South Africa 2010			Germany 2006			Korea/Japan 2002		

	Winning (n = 46)	Drawing (n = 28)	Losing (n = 46)	Winning (n = 48)	Drawing (n = 22)	Losing (n = 48)	Winning (n = 45)	Drawing (n = 28)	Losing (n = 45)
Variables related to attacking play									
**GS**	**2.1 ±1.2^ab^**	0.7 ±0.7	0.5 ±0.6	**2.2 ±1.1^ab^**	0.8 ±0.8	0.3 ±0.6	**2.2 ±1.3^ab^**	**1.1 ±0.7^b^**	0.4 ±0.7
**TS**	**16.0 ±5.5^ab^**	12.1 ±5.4	12.7 ±4.7	**14.3 ±4.9^ab^**	10.9 ±3.9	9.6 ±4.1	**12.4 ±4.5^b^**	10.7 ±3.7	9.8 ±4.0
SofT	8.9 ±3.8	8.1 ±4.0	8.8 ±3.4	6.6 ±3.4	6.4 ±2.5	5.8 ±2.8	5.8 ±3.0	5.7 ±2.7	5.4 ±2.7
**SonT**	**7.1 ±2.7^ab^**	3.9 ±2.2	3.9 ±2.0	**7.7 ±2.6^ab^**	4.6 ±2.9	3.8 ±2.2	**6.6 ±2.5^ab^**	5.0 ±2.0	4.4 ±2.5
**BP%**	**52.4 ±6.0^b^**	49.7 ±5.3	47.8 ±6.0	**52.4 ±7.2^b^**	50.0 ±4.6	47.5 ±7.2	49.8 ±7.0	50.0 ±7.2	50.2 ±7.0
OSc	2.4 ±2.0	2.4 ±2.0	2.1 ±1.5	3.5 ±2.7	2.6 ±2.8	2.8 ±1.8	2.9 ±2.6	3.0 ±2.4	2.6 ±1.7
**Fr**	16.0 ±5.8	15.3 ±3.5	13.8 ±4.5	**19.9 ±7.4^b^**	17.7 ±4.7	16.7 ±5.4	18.1 ±4.5	17.9 ±5.3	17.1 ±5.0
**C**	5.2 ±4.5	4.4 ±3.5	4.5 ±2.6	**5.8 ±3.1^b^**	5.6 ±3.0	4.2 ±2.3	5.2 ±2.7	4.9 ±2.8	5.8 ±1.7
Variables related to defence									
**TSr**	12.7 ±4.7	12.1 ±5.4	**15.9 ±5.4^ac^**	9.6 ±3.9	10.9 ±3.9	**14.3 ±4.9^ac^**	9.8 ±4.0	10.7 ±3.7	**12.4 ±4.5^c^**
SofTr	8.8 ±3.4	8.1 ±4.0	8.9 ±3.8	5.6 ±2.7	6.4 ±2.5	6.6 ±3.4	5.4 ±2.7	5.7 ±2.7	5.8 ±3.0
**SonTr**	3.9 ±2.0	3.9 ±2.2	**7.1 ±2.7^ac^**	4.1 ±3.1	4.6 ±2.9	**7.7 ±2.6^ac^**	4.4 ±2.5	5.0 ±2.0	**6.6 ±2.5^ac^**
OSc	2.3 ±1.6	2.4 ±2.0	2.5 ±2.1	2.8 ±1.8	2.6 ±2.8	3.4 ±2.7	2.6 ±1.7	2.7 ±2.0	2.9 ±2.6
**Fc**	14.1 ±4.8	**16.1 ±3.7^c^**	16.3 ±5.9	17.0 ±5.5	17.7 ±4.7	19.2 ±4.9	17.1 ±5.1	17.9 ±5.3	18.1 ±4.5
**Ca**	4.4 ±2.5	4.9 ±3.4	5.1 ±2.9	4.1 ±2.3	5.6 ±3.0	**5.8 ±3.1^c^**	5.8 ±3.2	4.8 ±2.9	5.2 ±2.7
YC	1.8 ±1.4	2.1 ±1.2	1.9 ±1.1	2.4 ±1.4	2.0 ±1.3	2.5 ±1.3	1.8 ±1.3	2.5 ±1.7	2.0 ±1.5
**RC**	0.02 ±0.1	0.1 ±0.3	**0.3 ±0.4^ac^**	0.08 ±0.3	0.3 ±0.6	0.2 ±0.5	0.1 ±0.25	0.1 ±0.3	0.2 ±0.5

GS is Goals scored, TS is Total shots, SofT is Shots off target, SonT is Shots on target, BP% is Ball possession (%), OSc is Off sides committed, Fr is Fouls received, C is Corners, TSr is Total shots received, SofTr is Shots off target received, SonTr is Shots on target received, OSr is Off sides received, Fc is Fouls committed, Ca is Corners against, YC is Yellow cards and RC is Red cards,

a Significantly different from drawing teams.

b Significantly different from losing teams,

c *Significantly different from winning teams*.

**Table 4:** Standardized coefficients from the discriminant analysis of match statistics for winning, drawing and losing teams from the whole sample of matches played in the last three World Cups

Variables	Function 1	Function 2
***Total shots***	.36*	−.37*
*Shots off target*	.06	.05
***Shots on target***	.62*	.65*
*Ball possession (%)*	.23	−.03
*Off sides committed*	.09	.04
*Fouls received*	.20	.02
*Corners*	.10	.09

***Total shots received***	−.37*	.37*
*Shots off target received*	−.07	.04
***Shots on target received***	−.56*	.66*
*Off sides received*	−.09	.13
*Fouls committed*	−.18	−.05
*Corners against*	−.10	.01
*Yellow cards*	−.06	.19
*Red cards*	−.23	.01

Eigenvalue	0.83	0.08
Wilks’ lambda	0.50	0.93
Canonical correlation	0.67	0.27
Chi-squared	236.43	26.32
Significance	0.00	0.02
% of variance	91.4	8.6

* SC discriminant value ≥.30

**Table 5:** Standardized coefficients from the discriminant analysis of match statistics for winning, drawing and losing teams in each of the three World Cups

	
	South Africa 2010 Function		Germany 2006 Function		Korea/Japan 2002 Function	
	1	2	1	2	1	2
Variables related to attacking play						

**Total shots**	−.25	.45*	.45*	−.16	−.50*	.19
Shots off target	−.01	.18	.11	.04	−.11	−.18
**Shots on target**	−.53*	.69*	.65*	−.33*	−.74*	.54*
**Ball possession (%)**	−.32*	.10	.31*	.02	.06	.01
**Off sides committed**	−.05	−.05	.11	−.18	−.11	−.36*
Fouls received	−.19	−.05	.22	−.08	−.18	−.17
**Corners**	−.10	.15	−.25	.18	.15	.59*

Variables related to defence						

**Total shots received**	.27	.37*	−.46*	−.22	.09	−.08
Shots off target received	.02	.18	−.14	.05	.11	−.16
**Shots on target received**	.56*	.53*	−.51*	−.43*	.01	.06
Off sides received	.05	−.03	−.11	−.18	.02	−.03
Fouls committed	.18	−.19	−.18	−.09	−.11	−.17
**Corners against**	.10	−.46*	−.25	.18	.06	−.08
Yellow cards	.03	−.22	−.02	−.28	−.04	−.07
**Red cards**	.31*	.16	−.15	.26	.32*	.33*

Eigenvalue	1.17	0.21	1.17	0.21	0.30	0.02
Wilks’ lambda	0.38	0.82	0.38	0.82	0.75	0.98
Canonical correlation	0.73	0.42	0.73	0.42	0.48	0.14
Chi-squared	107.66	21.50	104.64	20.82	31.78	2.28
Significance	0.00	0.04	0.00	0.106	0.02	0.97
% of variance	84.6	15.4	84.7	15.3	93.6	6.4

* SC discriminant value ≥.30
